# Vinegar Production from Corinthian Currants Finishing Side-Stream: Development and Comparison of Methods Based on Immobilized Acetic Acid Bacteria

**DOI:** 10.3390/foods10123133

**Published:** 2021-12-17

**Authors:** Iris Plioni, Argyro Bekatorou, Antonia Terpou, Athanasios Mallouchos, Stavros Plessas, Athanasios A Koutinas, Eleftheria Katechaki

**Affiliations:** 1 Department of Chemistry, University of Patras, 26504 Patras, Greece; plioni@upatras.gr (I.P.); a.a.koutinas@upatras.gr (A.A.K.); 2 Department of Agricultural Development, Agri-Food, and Natural Resources Management, School of Agricultural Development, Nutrition & Sustainability, National and Kapodistrian University of Athens, 34400 Athens, Greece; aterpou@agro.uoa.gr; 3 Department of Food Science and Human Nutrition, Agricultural University of Athens, 75 Iera Odos, 11855 Athens, Greece; aMallouchos@aua.gr; 4 Laboratory of Food Processing, Faculty of Agriculture Development, Democritus University of Thrace, 68200 Orestiada, Greece; splessas@agro.duth.gr; 5 Agricultural Cooperatives Union of Aeghion S. A., Korinthou 201, 25100 Aeghion, Greece; elkatehaki@pesunion.gr

**Keywords:** vinegar, Corinthian currants, finishing side-stream, acetic acid bacteria, immobilized cells, volatiles, phenolic content, antioxidant capacity

## Abstract

Fruit wastes and side-streams can be used for vinegar production to create added value for the agri-food sector and enhance farmer incomes and local economies. In this study, methods for vinegar production by wild and selected acetic acid bacteria (the quick starter *Acetobacter aceti* and the acid-resistant *Komagataeibacter europaeus*), free (FC) and immobilized (IC) on a natural cellulosic carrier, are proposed using sweet wine made from the industrial finishing side-stream (FSS) of Corinthian currants as raw material. The results showed all cultures can produce vinegar with 46.65 ± 5.43 g/L acidity, from sweet FSS wine containing 5.08 ± 1.19% alcohol. The effect of immobilization was more obvious in the case of the selected culture, presenting better acetification efficiency, both fresh and after cold storage for 2 months. The vinegars had an antioxidant capacity of 263.5 ± 8.4 and 277.1 ± 6.7 mg/L (as ascorbic acid) and phenolic content 333.1 ± 12.0 and 222.2 ± 2.9 mg/L (as gallic acid) (for FC and IC, respectively). They also had a rich volatilome (140 compounds identified by SPME GC-MS), with higher percentages of esters identified in vinegars made by IC. The results are encouraging for vinegar production with IC of a mixed *A. aceti* and *K. europaeus* culture.

## 1. Introduction

Vinegar is a liquid product, which contains 4% or more acetic acid and is used or consumed as is or as a food ingredient [1]. It is commonly produced by two-stage fermentation systems: (a) alcohol fermentation of carbohydrate-containing substrates, and (b) oxidation (“acetification”) of the produced alcohol into acetic acid. Commonly used raw materials for vinegar production are fruit such as apples (cider vinegar) and grapes (wine vinegar), other plant sources such as rice, malt, etc., (cereal vinegar), sugar cane, and any carbohydrate-containing material [1,2]. Vinegar is available worldwide at a variety of quality, types, and prices: from cheap distilled or synthetic vinegar and common wine/cider vinegars to very expensive traditional balsamic products [1]. Its flexibility and variety of applications have placed it among the most valuable food products, with an estimated global market value of ~2.25 billion US$ in 2020, expecting to reach ~2.55 billion US$ by 2026, considering the uncertainties caused by the COVID-19 pandemic [3].

Vinegar is produced by the action of acetic acid bacteria (AAB), which are commonly found in the raw materials used for vinegar production. The currently recognized AAB genera belong to the family of *Acetobacteraceae*, with most species found in vinegar belonging to *Acetobacter* and *Gluconacetobacter* [2]. In industrial vinegar production, three main methods can be distinguished: the slow, traditional *Orleans* (or *French*) method (slow, surface acetification carried out in wooden barrels), the fast *Generator* method (acetification by forced aeration in the presence of wood shavings or other inert material), and the rapid *Submerged* method (batch fermentation with forced aeration). Despite the vast scientific knowledge gained on the production of vinegar, the industry mainly uses submerged fermentation (SmF) techniques, in which the main vessel is the *acetator* (acetification reactor). Acetification can be spontaneous, using culture from a previous batch, or by adding selected cultures to secure large-scale vinegar production, in terms of yield, safety, process stability, short fermentation time, product losses, and undesirable characteristics caused by uncontrolled fermentation. However, the use of selected AAB starters is still a long way from being a common practice [4].

On the other hand, molecular methods have helped identify AAB strains with appropriate traits. According to [4], *Acetobacter* spp. predominate at low acetic acid concentrations. When acetic acid exceeds 5%, species such as *Komagataeibacter europaeus* or *Gluconacetobacter intermedus* are predominant. Generally, a process that involves a mixed culture of a “quick starter” (e.g., *Acetobacter pasteurianus* or *Acetobacter aceti*) and one with high resistance to acetic acid (e.g., *K. europaeus*), is considered the best practice to ensure good performance and evolution of the fermentation. The final quality of the product also depends on the suitable culture selection [4].

Among other innovative methods that have been proposed to improve fermentation efficiency, cell immobilization techniques have been successfully applied at both research and industrial level for food production including vinegar [5,6,7,8,9]. However, most modern vinegar factories produce vinegar by SmF processes that last 4 to 5 weeks. Therefore, there is still a need for developing appropriate technologies for fast and cost effective production of vinegar, but not at the expense of quality. Cell immobilization is particularly interesting as it can improve the fermentation process by increasing the culture density in the reactors (and consequently the fermentation rates and yields), allowing culture reuse and facilitating continuous operation and recovery of the products. Materials that are cheap, easily available, and inert, such as wood shavings, cellulose, hydrocolloid gels, etc., can be used as culture immobilization carriers. However, industrial application of immobilized biocatalysts is limited due to issues related to the design of proper large-scale processes, handling, contamination, etc., as well as due to the difficulty of industries in adapting novel technologies [5,10].

Regarding alternative raw materials for vinegar production, cheap substrates such as fruit and vegetable surpluses, agro-industrial by-products, side streams, and wastes [9] can be used. Their utilization can enhance the farmers’ incomes and the local economies, especially in developing countries, and create added value for the agri-food sector in general. In this study, the industrial finishing side-stream (FSS) of premium quality Corinthian currants is proposed for added value vinegar production. A Corinthian currants processing company produces FSS that accounts for about 5–6% of the initial material, which differs from the marketable product mainly in the size of the berries and the presence of seeds. FSS contains high amounts of fermentable sugar (~70% glucose and fructose, with traces of saccharose), and has a rich volatilome, and increased antioxidant capacity, and total fat and phenolic contents, compared to the marketable currants [10]. It is commonly used in low-price vinegar or syrup production. Its use for winemaking, at low temperatures by free and immobilized yeast cells, combined with baker’s yeast production, was recently proposed by our research group [10]. The wines contained no methanol and had higher levels of terpenes and volatile compounds (fermentation products), compared to FSS. A technoeconomic analysis that was based on the design of an integrated industrial plant for simultaneous wine and baker’s yeast production to utilise all sugar contained in FSS, showed that the investment would be realistic and profitable [10].

In this study, innovative fermentation methods for the production of vinegar are proposed, using sweet FSS wine as raw material, and immobilized or free cells of mixed *Acetobacter aceti* and *Komagataeibacter europaeus* and wild vinegar cultures as starters. The fermentation efficiency of these cultures after 2 months of cold storage was also evaluated.

## 2. Materials and Methods

### 2.1. Chemicals 

NaOH (Lach-Ner, Neratovice, Czech Republic). Std. 0.1 M NaOH solution, (NH_4_)_2_SO_4_, MgSO_4_·7H_2_O, saccharose, and fructose (Chem-Lab, Zedelgem, Belgium). Glucose, methanol, ethanol, and phenolphthalein (Fisher Scientific, Loughborough, UK). Yeast extract (Duchefa Biochemie, Haarlem, Nederlands). Glucose monohydrate for microbiology, KH_2_PO_4_, and 2-propanol (Merck, Darmstadt, Germany). 2,2-Diphenyl-1-picrylhydrazyl radical (DPPH), and gallic acid (Sigma-Aldrich, St. Louis, MO, USA). Acetic acid (Carlo-Erba, Val de Reuil, France). Folin–Ciocalteu reagent (Scharlab S. L., Sentmenat, Spain). Anhydrous Na_2_CO_3_ (Penta, Prague, Czech Republic). Ascorbic acid (Fluka, Sigma-Aldrich, Buchs, Switzerland). Peptone and mannitol (Conda, Madrid, Spain). C8-C24 n-alkanes (Niles, IL, USA).

### 2.2. Raw Materials, Microorganisms, and Media

The FSS used in this study was supplied by the Agricultural Cooperatives’ Union of Aeghion S.A. (Aigio, Greece) from the crop years 2017–2019. It was initially extracted by maceration with hot water at 70 °C and used for sweet winemaking by partial alcohol fermentation [10]. The received extract contained 272 g sugar/L and was used without pasteurization or sulphite addition. 

The cryotolerant and alcohol resistant yeast strain *Saccharomyces cerevisiae* AXAZ-1, isolated and available at the University of Patras, was used for winemaking by the alcoholic fermentation of the FSS extract. It was grown at 30 °C in a sterile medium containing: (g/L) glucose 20, yeast extract 4, (NH_4_)_2_SO_4_ 1, KH_2_PO_4_ 1, and MgSO_4_·7H_2_O 5 [11].

The quick fermentation starter *A. aceti* (DSM No. 3508), isolated from alcohol-turned-to-vinegar fermentation, was supplied by the Leibniz Institute Deutsche Sammlung von Mikroorganismen und Zellkulturen GmbH, German Collection of Microorganisms and Cell Cultures (DSMZ), and was used for the vinegar production experiments. It was initially grown on solid medium containing (g/L) yeast extract 5, peptone 3, mannitol 25, and agar 12, in deionized water, for 72 h at 30 °C. Then, it was further grown for 72 h at 30 °C in a sterile medium with the same composition without agar addition. 

The acetic acid resistant strain *K. europaeus* (DSM No. 6160) was also supplied by DSMZ and was used for the vinegar production experiments. It was initially grown on solid medium containing (g/L): yeast extract 2, peptone 3, glucose 5, agar 10, acetic acid (40 mL/L), and ethanol (30 mL/L), in deionized water, for 72 h at 30 °C. Then, it was further grown at 30 °C in a sterile medium with the same composition without agar addition. Ethanol and acetic acid were aseptically added through a bacteriostatic filter.

All cultures were harvested by centrifugation at 5000 rpm for 10 min on a Sigma 3K12 Bioblock Scientific centrifuge (Sigma Larborzentrifugen GmbH, Osterode, Germany). 

The wild AAB culture in liquid form (*mother of vinegar*) was supplied by a local vinegar producer in Patras, Greece. It was stored at 4 °C and was also used for the vinegar production experiments. 

### 2.3. Vinegar Production from FSS

#### 2.3.1. Alcoholic Fermentation for Sweet Winemaking 

For winemaking, partial alcoholic fermentation of the FSS extract was carried out by *S. cerevisiae* AXAZ-1 (16.4 g harvested biomass per 400 mL of FSS extract). The fermentation was monitored until a sweet wine with about 10% *v*/*v* alcohol and 9–10% *w*/*v* residual sugar was obtained. The wine was diluted each time at a ratio of 1:1 with water to obtain the vinegar fermentation substrate, which finally contained 3.59 ± 0.60% *w*/*v* sugar and 5.08 ± 1.19% *v*/*v* alcohol (Table 1).

#### 2.3.2. Mixed AAB Starter Preparation 

After several preliminary acetification experiments, which did not allow proper vinegar production due to indications of spoilage, the following successful acetification protocol was adopted: The harvested *A. aceti* and *K. europaeus* cultures (at a ratio of 1:1) were mixed with the sweet FSS wine and water at a volumetric proportion of 25:50:25 (inoculum:wine:water), producing an initial starter (mother of vinegar). To increase the mother of vinegar volume, an equal volume of sweet FSS wine was added before ethanol was completely exhausted by the bacteria, and this process was repeated until a final volume of 400 mL was obtained. This mother of vinegar (mixed AAB culture), immobilized or free, was used to carry out the subsequent vinegar fermentation, after its preparation (fresh) and after 2 months of cold storage at 4 °C. Cold storage of each culture was performed after it had completed the 3-batch acetification cycle. In the case of immobilized cells, they were collected, placed in a glass container and covered with a quantity of vinegar/wine (1:1). In the case of free cells, they were harvested by centrifugation and redispersed in a small quantity of vinegar/wine (1:1). After cold storage, the vinegar/wine solution was drained, and the immobilized or free cells were used again for vinegar production. 

#### 2.3.3. Vinegar Production Using Immobilized or Free AAB Cultures

**Figure 1:**
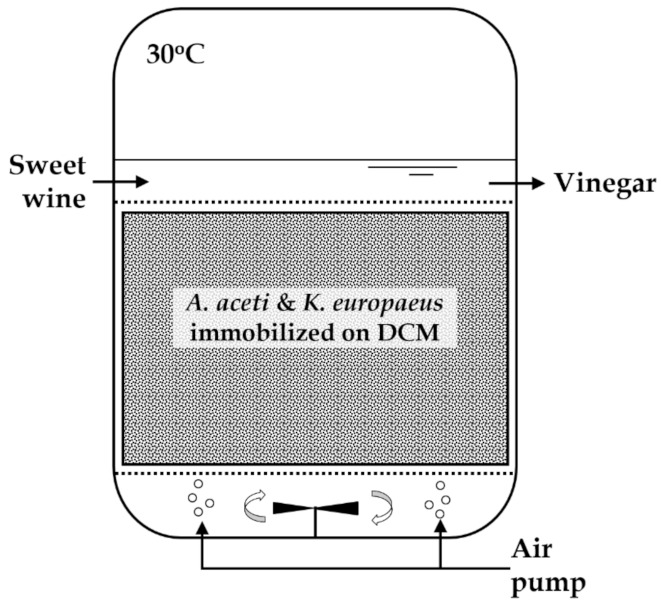
Bioreactor configuration for vinegar production from sweet FSS wine using immobilized acetic acid bacteria.

Delignified cellulosic material (DCM) was used as the cell immobilization carrier. It was prepared by boiling pine sawdust with NaOH solution as described before [10,11]. For vinegar production, acetification of 400 mL of the sweet FSS wine with 400 mL of the mixed AAB culture, was carried out at 30 °C, 250 rpm agitation speed, and 500 cm^3^/min air supply (Elite 803 air pump; Rolf C. Hagen UK Ltd., Castleford, UK) (Figure 1). 

Amounts of 100 g DCM, 2.16 g (NH_4_)_2_SO_4_ and 0.48 g KH_2_PO_4_ were added to the mixture. The fermentations were carried out until vinegar with a titratable acidity of about 40 g/L was produced. At this point, 400 mL of produced vinegar was removed and 400 mL FSS wine, 2.16 g (NH_4_)_2_SO_4_, and 0.48 g KH_2_PO_4_ were added.

In the same manner, vinegar production was carried out using, immobilized and free cells of both the wild AAB culture and the selected, mixed *A. aceti* and *K. europaeus* culture. For comparison, vinegar fermentations using free cells (wild or selected AAB cultures), without the addition of DCM, were also carried out. Moreover, the fermentation efficiency of the cultures (free and immobilized) after 2 months of cold storage (4 °C), was also studied. Vinegar made by the mixed *A. aceti* and *K. europaeus* culture are from this point on abbreviated as FCM and ICM, and those made by the wild culture as FCW and ICW, for free and immobilised cells, respectively. Correspondingly, these cultures after 2 months of storage are abbreviated as FCM-2M, ICM-2M, FCW-2M, and ICW-2M. 

The produced vinegars were analysed for ethanol, methanol, residual sugar, titratable acidity, volatiles, total phenolic content, and antioxidant capacity. Differences between the means of various data populations were analysed by t-test or One-Way ANOVA (at the *p* = 0.05 level) on Origin 6.0 Professional (Microcal Software, Inc., Northampton, MA, USA). 

### 2.4. Analytical Methods 

#### 2.4.1. Total Acidity (TA) 

TA was determined by titration of 10 mL of the sample with standard 0.1 M NaOH solution with phenolphthalein as indicator, expressing the results as g acetic acid/L of vinegar [7].

#### 2.4.2. Ethanol and Methanol

Ethanol and methanol were determined as described in [10]. In brief, a Shimadzu GC-8A gas chromatography system was used, equipped with a Teknokroma column, a flame ionisation detector (FID), and a C-R6A Chromatopack integrator. The column oven was programmed at 100–130 °C, increasing at a rate of 10 °C/min. The injection port and FID temperatures were both set at 210 °C. The sample injection volume was 2 μL, and 2-propanol a dilution of 0.5 % *v*/*v* was used as internal standard (IS) [10].

#### 2.4.3. Sugars 

Sugars were also determined as described in [10], by high-performance liquid chromatography (HPLC). A Shimadzu LC-9A HPLC system was used with a DGU-2A degassing unit, a Nucleogel Ion 300 OA column, a CTO-10A column oven set at 33 °C, an LC-9A pump, and a RID-6A refractive index detector. The mobile phase used was 0.017 M H_2_SO_4_ solution at a flow rate of 0.55 mL/min. The sample dilution was 1% *v*/*v*, the injection volume was 40 μL, and 1% *v*/*v* 2-propanol solution was used as IS. Determinations were based on standard curves of fructose, glucose, and sucrose [10]. 

#### 2.4.4. Volatiles

The volatile profile of vinegar made by free and immobilized cells of both types of cultures, was analysed by gas chromatography/mass spectrometry (GC/MS) with headspace sampling by solid-phase micro-extraction (SPME; fibre DVB/CAR/PDMS, 2 cm; Sigma-Aldrich, part of Merck KGaA, Darmstadt, Germany), as described by [10] with minor modifications. Specifically, 1 mL of vinegar, 8.5 mL of pre-boiled deionized water, 1 g of (NH_4_)_2_SO_4_, and 500 µL of a 1000 mg/L 1,4-dioxane solution as IS, were added in a 20 mL glass vial. The vial was then sealed with a silicone septum and was immersed in a water bath (40 °C) for 5 min with stirring (250 rpm, stir speed 5, Thermodyne-Nuova II). The SPME fibre was then exposed to the headspace above the liquid in the vial for 30 min. For desorption and chromatographic separation of the volatiles, the fibre was exposed in the injection port of the gas chromatograph (GC), in split mode (split ratio 1/10), at 240 °C for 5 min. The injection port carried an SPME liner (Sigma Aldrich) with 0.7 mm i.d. Before each analysis, the fibre was exposed in the injection port of another GC for 5 min at 250 °C, to remove any volatile residues. The GC instrument (GCMS-QP2010 Ultra, Shimadzu Inc., Japan) was equipped with a DB-Wax capillary column (30 m, 0.25 mm i.d., 0.25 μm film thickness, Agilent, Santa Clara, CA, USA), incubated at the following temperature program: 40 °C for 5 min, increased to 180 °C at a 5 °C/min rate, then increased to 240 °C at a 30 °C/min rate, and held at 240 °C for 5 min. The carrier gas was He at a linear velocity of 36 cm/sec. The mass spectrometer (MS) was operated in electron ionization mode, at an ionization energy of 70 eV and 4 at 0–300 m/z mass scan range. The source and interface temperatures were set at 200 °C and 240 °C, respectively.

For the identification of volatiles, the following were compared: (i) Retention indices (RI) based on the homologous series of C8-C24 n-alkanes with those of available authentic compounds and those available in the NIST14 library (NIST, Gaithersburg, MD, USA), and (ii) MS data with those of reference compounds and data available in the NIST14 library. The identification was effected using the software GCMS Solution (ver. 4.30; Shimadzu), Amdis (ver. 2.72; NIST), and NIST MS Search (ver. 2.2; NIST), and the reliability of identification (RID) was set at 3 levels A, B, and C (A: RI and MS data agreement with those of authentic compounds assayed under the same conditions; B: agreement of RI (ΔRI < 20) and MS data (similarity match > 900); C: at least ΔRI < 20 or MS data similarity match > 800. The content of volatile compounds is presented as normalized peak areas %, estimated by determining the percentage of the area corresponding to an Amdis component relative to the sum of the areas of all components [10]. 

#### 2.4.5. Total Phenolic Content (TPC) and Antioxidant Capacity (AC)

TPC analysis was carried out as described by [10]. Specifically, in 10 mL volumetric flasks, 0.1 mL of sample, 5 mL of water, and 1 mL of the Folin–Ciocalteu reagent were added and the flasks were left for 30 min in the dark. Subsequently, 1 mL of 7.5% *w*/*v* Na_2_CO_3_ solution was added, the volume was fixed to 10 mL, and the flask was left again for 30 min in the dark. Finally, the absorbance of the mixture was measured at 725 nm on a Jasco V-630 UV-vis spectrophotometer. The TPC was determined based on a gallic acid calibration curve and blank samples, and it was expressed as mg gallic acid equivalents (GAE). 

The AC of the vinegar, as radical scavenging capacity, was also evaluated as described by [10], based on the decrease in absorbance at 517 nm of a 2,2-diphenyl-1-picrylhydrazyl radical (DPPH) solution in methanol against aqueous methanol solution as blank. Specifically, 3 mL of 137.6 μM DPPH solution and various amounts of sample (0.05–1.00 mL) were added in 8 test tubes, and methanol was added to fix the volume to 4 mL. The tubes were left for 30 min in the dark and then the absorbance at 517 nm was obtained. The AC was determined based on an ascorbic acid calibration curve and expressed as mg ascorbic acid equivalents (AAE). 

#### 2.4.6. Scanning Electron Microscopy (SEM) 

The surface of the immobilized biocatalysts (DCM with entrapped AAB cells) was observed by SEM. The samples were oven-dried at 30 °C for 3 days and then stored in a desiccator until the SEM study. Prior to analysis, the samples were coated with gold (Baltec MED 020 high-vacuum coating system) and observed on a Jeol JSM-6300 SEM instrument available at the Laboratory of Electron Microscopy and Microanalysis of the University of Patras. 

## 3. Results and Discussion

In this study, the use of sweet wine produced from the side-stream (FSS) generated during the industrial standardization of premium quality Corinthian currants is proposed as raw material for vinegar production by a selected, mixed *A. aceti* and *K. europaeus* culture, and a wild vinegar culture, free (FCM and FCW, respectively) and immobilised (ICM and ICW, respectively) on a food-grade natural cellulosic material. The acetification efficiency of these cultures after 2 months of cold storage was also studied, to evaluate the possibility of culture reuse in case the production process must be ceased. Another innovation is the use of sweet wine made from FSS instead of dry wine, to evaluate the effect of partially fermented sources on the growth and fermentation efficiency of the AAB cultures. Following, the results of the vinegar fermentation kinetics, phenolic content, antioxidant capacity and volatilome are presented. 

### 3.1. Immobilization of the AAB Cultures

**Figure 2:**
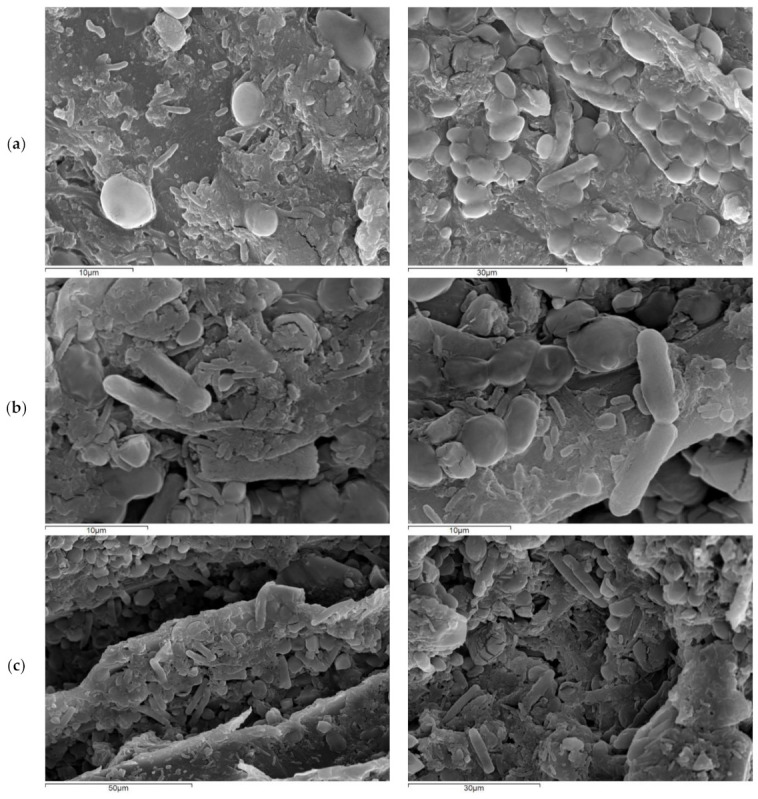
SEM images of the immobilized biocatalysts on DCM during the acetification of sweet FSS wine. (**a**): wild culture (ICW). (**b**,**c**): *A. aceti* and *K. europaeus* culture (ICM).

In Figure 2a, the SEM images of the immobilized species during ICW vinegar fermentation are presented. Bacterial cells with lengths below 2 μm correspond with the morphology of the AAB species. According to [12], AAB cell length slightly decreases when ethanol is present, and significantly decreases (from an average of 1.4 to 1.0 μm) when the acetic acid content in the culture medium reaches 1.7%. It remains stable during the rest of the acetification process. Ellipsoidal cells ∼5 μm in diameter, correspond with the morphology of budding yeasts, such as *Saccharomyces cerevisiae* [13], while cylindrical rod-shaped cells with lengths above 7 μm that divide by medial fission correspond with the morphology of *Schizosaccharomyces* spp. [14]. 

In Figure 2b,c the SEM images of the immobilized species during ICM vinegar fermentation are presented (*A. aceti* and *K. europaeus*). In both cases, a large number of cells seem to be attached to the surface of the DCM carrier.

The presence of species such as *Schizosaccharomyces* yeasts in the vinegar produced by the selected culture can be explained by the fact that the FSS extract used to produce the wine was not previously pasteurized, neither SO_2_ was added, thus preserving its natural microflora. During the subsequent alcohol fermentation and acetification processes, this microflora was enriched and carried to the final products. Since, the study aimed at developing processes that are not very complex and are suitable for small to medium scale application, the work focused mainly on determining the acetification efficiency of the different biocatalysts as well as their effect on major vinegar attributes such as its volatilome, phenolic content, and antioxidant capacity. More detailed work, including microbiological analysis, should be done in future works based on vinegar production by specific cultures.

### 3.2. Acetification Process Parameters 

DCM, a cheap, readily available, and inert biomaterial, was used as an immobilization carrier for the microorganisms involved and as a promoter of the FSS wine acetification process [15]. The composition of FSS and its aqueous extract, as well as its use for dry winemaking using *S. cerevisiae* AXAZ-1 immobilised on DCM, were previously reported by [10]. One of the novelties of the methods presented in this study is that the alcoholic fermentation was partial until a sweet FSS wine was obtained, therefore the subsequent acetification was carried out in the presence of sugars in the substrates. 

In Table 1, parameters of the sweet wine acetification process using the wild and the selected AAB cultures, free and immobilized on DCM and after 2 months of cold storage, are presented, as means of 3 repeated acetification batches. Analysis of variance (One-way ANOVA at the *p* = 0.05 level) between the data populations of the 3 batches, showed that the means of sugar, ethanol, and acidity concentrations were not significantly different (*p* > 0.05), with the exception of the ethanol levels for FCM, FCW-2M, FCM-2M, and ICW-2M (*p* < 0.05). For FCM-2M the means of all parameters of the 3 batch fermentations were significantly different. 

Generally, it can be observed that all cultures, free or immobilised, can produce vinegar with 46.65 ± 5.43 g/L levels of acidity (as g acetic acid/L) from sweet wine with 5.08 ± 1.19% *v*/*v* alcohol concentration. The differences observed in the initial sugar and ethanol concentration of the substrates are due to the production method used, i.e., an amount of the produced vinegar was decanted and the remaining liquid served as the starter of the next batch, as in the case of the common industrial SmF processes. This is a technological feature that needs further investigation. However, this is a low-volume bench-scale experiment and these variations are not easy to control compared to the commonly used 8–10 tn, or higher capacity, industrial acetator vessels. Moreover, the final industrial vinegar products are commonly diluted with water to the desired acidity before packaging. The most important requirement in the vinegar industry is to keep the acetators functioning because acetification cease is usually detrimental for the AAB and the whole process must restart. 

**Figure 3:**
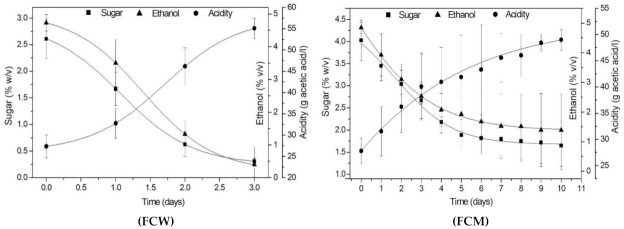

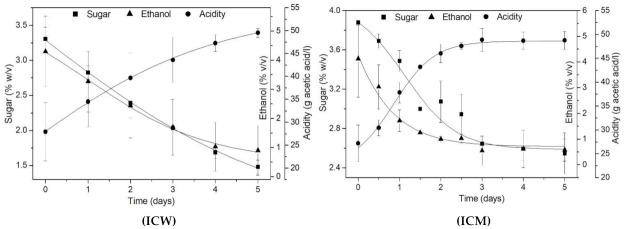
Kinetics of the acetification of wine at 30 °C, using wild AAB culture, free (FCW) and immobilized (ICW), and the selected, mixed AAB culture (*A. aceti* and *K. europaeus*), free (FCM) and immobilized (ICM) (symbols are the mean values plus standard deviations of the experimental data from 3 batches; curves are sigmoidal Boltzmann’s model fits).

**Figure 4:**
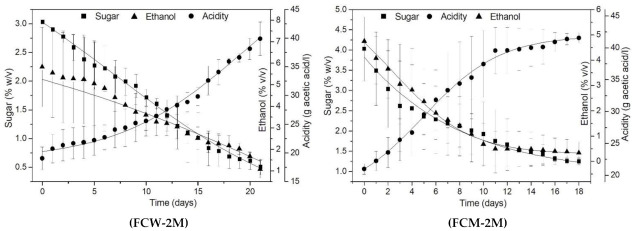

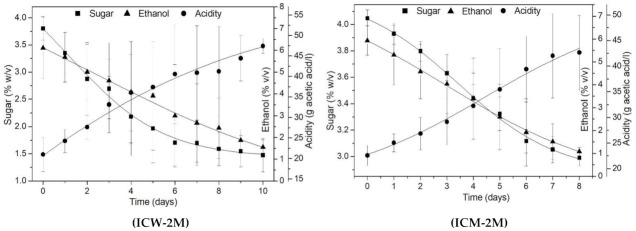
Kinetics of the acetification of wine at 30 °C, reusing the wild AAB culture, free (FCW-2Μ) and immobilized (ICW-2Μ), and the mixed AAB culture (*A. aceti* and *K. europaeus*), free (FCM-2Μ) and immobilized (ICM-2Μ), after 2-months of cold storage at 4 °C (symbols are the mean values plus standard deviations of the experimental data from 3 batches; curves are sigmoidal Boltzmann’s model fits).

Figure 3 and Figure 4, present the kinetics of the acetification of the sweet wine at 30 °C using the wild or selected cultures, free, immobilized, and after 2 months of cold storage. The average values of 3 batches plus standard deviations are illustrated for the evolution of sugar, ethanol, and acidity levels during the process. Best curve fitting of the data was done using Boltzmann’s sigmoidal function, on Microcal Origin 6.0 software, achieving very high coefficients of determination (R^2^ > 0.99) for the majority of curve fits (Tables S1 and S2, Figures S1 and S2).

Specifically, Figure 3 illustrates the acetification kinetics of the FSS sweet wine using the fresh, free and immobilised, wild AAB cultures (FCW, ICW) and the mixed *A. aceti* and *K. europaeus* cultures (FCM, ICM). Comparing the data from Table 1 and Figure 3, it can be observed that it is possible to produce vinegar from sweet FSS wine in repeated acetification batches using the same culture, by all 4 treatments. The acetification times ranged from 3–5 days to 2–9 days for the fresh wild and mixed cultures, respectively, free and immobilised. The effect of cell immobilization on the acetification kinetics was more obvious in the case of the selected, mixed culture. In a similar manner, comparing the data from Table 1 and Figure 4, it can be observed that in the case of cultures stored for 2 months at 4 °C, the acetification times varied from 10–20 days to 7–11 days for the wild and mixed cultures, respectively, free and immobilised. In this case, the effect of cell immobilization was obvious for both types of cultures (wild and mixed), which seemed to readapt fast to the acetification conditions after storage and complete the process in shorter times. 

In the case of immobilised cells, another advantage is that the recovery of the biocatalyst is easier since the whole biocatalyst remains in the bioreactor, while the vinegar can be easily decanted as soon as the fermentation completes and has a better clarity compared to the vinegar made by free cells. 

The literature indicates that the acetification can take place within 30 h depending on the desired acetic acid content [16]. Other studies have reported that the acetification times to produce vinegar from various fruit using immobilized AAB ranged from 5–28 days [7,17,18,19]. The acetification times observed in this study were 3 and 20 days using free cells, and 2 and 10 days using immobilized AAB cells (before and after storage, respectively), to obtain acidity levels in the range 41–50 g/L. Therefore, the acetification times with immobilized cells on DCM were shorter. However, comparisons with data from other studies may be quite ineffective, due to the different method parameters applied (bioreactor design, aeration intensity and air dispersion, composition of the substrate, and the AAB cell density in the bioreactors).

The cultures were stored in cold to evaluate their acetification efficiency after storage at low temperature, in the case that the vinegar production process has to be ceased. The optimum growth temperature for AAB is 25–30 °C. Growth has been observed at lower temperatures but at very low rates [2,20]. Cold storage of the cultures for 2 months reduced, as expected, the activity of both free and immobilized cells during the first fermentation batch, but the rate of the subsequent batches remained at satisfactory levels. Therefore, the process presents an improvement as the fermentation bathes proceed, ensuring the functional stability of the acetator. This improvement may be attributed to the increased viability of the immobilized cells and their better adaptation to the system, as indicated in previous studies reporting increased fermentation productivity induced by cell immobilization (on DCM or other carriers) [6,11,21].

Thus, it can be concluded that the use of sweet wine (partially fermented) as raw material in the production of vinegar is possible using both wild and selected AAB cultures. The cultures were able to convert both the alcohol and the sugar contained in the FSS wines (into acetic acid, cell mass, and other metabolites), indicating that although the presence of sugars seems to promote the activity of AAB, the proposed method is not suitable for the production of new types of “sweet” vinegar. Therefore, for sweet vinegar production from FSS (e.g., resembling balsamic vinegar), FSS extract or syrup should be added post-acetification. 

### 3.3. Phenolic Content and Antioxidant Capacity

The composition of vinegar is influenced by the raw material as well as the acetification process. Specifically, phenolic components affect both vinegar quality (colour, taste) and nutritional value; mainly its antioxidant capacity among other health benefits [22,23]. Table 2 presents the results of the analysis of TPC and AC of the vinegar made from the FSS sweet using the mixed *A. aceti* and *K. europaeus* culture, free and immobilized on DCM. 

The TPC was higher in vinegar made with free cells. This difference is likely due to the presence of DCM in the case of immobilized cells, which probably adsorbs phenolic compounds on its surface as also shown in previous studies [6]. However, the TPC values were similar to those of white vinegar (109–224 mg GAE/L) [23,24]. On the other hand, although the TPC content was significantly different, the AC was similar for both types of vinegar (Table 2). This may be due to either the different oxidation states of the phenolic compounds present in the vinegar or the presence of other metabolites with antioxidant properties produced or converted at different rates by the different biocatalyst types (free or immobilized cells).

### 3.4. Volatiles

The composition and complexity of the volatile profile of vinegar, and therefore its aroma, is directly related to the quality and value of vinegar products. The main factors that determine the volatile fraction in vinegar are the raw material and the production process (alcoholic fermentation, acetification, the ageing process, etc.). So far, the effect on the volatile profile of vinegar of the microorganisms involved in the production process has little been studied [25]. Despite the significant changes caused by the biological processes of vinegar production, the products retain a volatile fraction from the raw material. Moreover, the strong air supply during production can lead to loss of volatile compounds, while the ageing processes to which the high-quality vinegar are usually subjected also contributes to the complexity of aroma and the added value of the products [25].

Volatiles in vinegar mainly include alcohols, organic acids, ethyl and acetate esters. Depending on the type of vinegar, various types and amounts of aldehydes, ketones, acetals, lactones, terpenes, volatile phenols and pyrazines may also be found. Typical volatile compounds of vinegar, such as acetic acid, acetoin, and ethyl acetate, are usually found in higher proportions in the volatile fraction than other compounds. In aged products, acetoin and ethyl acetate can reach amounts higher than 1000 and 3500 mg/L, respectively [25,26,27]. As in the case of FSS and dry wine made from FSS [10], the aroma profile of the FSS vinegar produced in this study was analysed by SPME GC-MS. A total of 140 compounds were detected, including 26 esters, 7 lactones, 25 alcohols, 13 organic acids, 37 carbonyl compounds, 13 terpenes, 8 hydrocarbons, and 11 other compounds (mainly pyrazines) (Table 3). The origin of these compounds (raw material, fermentation, etc.), and their effect on aroma were recently discussed in detail by [10]. The differences observed among the different products (FSS, dry wines and vinegar) are mainly quantitative.

The three major esters identified were ethyl acetate (30–46% of the total volatile fraction), isoamyl acetate (4–20%) and 2-phenylethyl acetate (1.6–6%), followed by isobutyl acetate, ethyl isovalerate, ethyl hexanoate, and ethyl octanoate at lower percentages. The percentage of esters in the vinegar made by immobilized cells was higher (62%) compared to those made by free cells (41–55%).

The main alcohols found in the products were ethanol, 3-methyl-1-butanol, and 2-phenylethanol. In general, alcohols were more abundant in the vinegar produced by free cells of the wild AAB culture (FCW).

Of the volatile organic acids, that were generally found at low concentrations (<0.01%), apart from acetic acid, mainly isovaleric, 2-methylbutyric, hexanoic, and octanoic acids (>0.1%) were detected.

The major carbonyl compounds were acetaldehyde, diacetyl, acetoin, and furfural. Acetaldehyde is an intermediate in the catabolism of C-sources by yeasts and AAB. Specifically, it is formed by the oxidation of ethanol and pyruvate [28]. Acetoin is also produced in significant quantities during the acetification and is often used as an indicator of the biological origin of vinegar, as its synthesis is related to the metabolism of AAB.

The major terpene compounds identified were D-limonene, linalool, α-terpineol, β-damascenone, p-cymen-8-ol and trans-geralynacetone. Thujone (>0.01%) was also found at higher percentages in the vinegar compared to the raw material (FSS). The same was observed in the case of dry wine made from FSS [10]. It is a monoterpene ketone, which is usually related to the presence of weed impurities, such as *Tanacetum vulgare* L. (tansy) commonly growing in vineyards [10,29]. However, the fact that it was found in all vinegar samples and dry wines made from FSS, indicates a correlation with either the fermentation process or the presence of grape seeds in FSS and should be further investigated by analysis of the FSS, other parts of the vine plant, and the seedless commercial Corinthian currants (fresh and dried), from which the FSS was generated. 

With respect to lactones, 4 γ-lactones, β-angelica lactone, and δ-octalactone were identified, as well as 2-hexene-1,4-lactone, which has not been previously reported in grapes, wines or vinegar.

In total, fewer volatiles were detected in the FSS vinegar compared to the dry FSS wines (160 compounds) previously reported by [10]. However, several compounds were only detected in the vinegar and the FSS analysed in this study: ethyl formate, benzyl acetate, 3-methyl-2-buten-1-ol, formic acid, 3-methyl-2-butanone, 2-methyl-2-butenal, hydroxyacetone, acetoin acetate, 1-hydroxy-2-butanone, phoracanthal, acetophenone, 2-acetylpyrrole, 2-hydroxyacetylfuran, eucalyptol, L-menthol, 4-ketoisophorone, p-cymen-8-ol, γ-octalactone, 2,6-dimethylpyrazine, 2-ethyl-6-methylpyrazine, 2,3,5-trimethylpyrazine, 2,3-dimethyl-5-ethylpyrazine, 2,3,5,6-tetramethylpyrazine (ligustrazine), and benzothiazole (Table 3). Among these compounds, the benzenoids and terpenoids originate from the phenylpropanoid/benzenoid pathway and the terpenoid pathway in plants, respectively [10,30]. A wide range of acids, aldehydes, ketones, alcohols and esters are present either as lipid degradation products of plant metabolites or as microbial metabolites generated during the fermentation processes involved in vinegar production. Pyrazines, furans, and benzothiazole are known products of the interactions of amino acids and sugars (Strecker degradation and Maillard reaction). They commonly occur in raisins due to the drying process [10,30,31]. However, compounds such as pyrazines may also be generated by de novo biosynthesis from microbial cultures [30]. Benzothiazole has also been reported as a result of the thermal processing of Chinese vinegar [32]. Ligustrazine has been associated with the antioxidant, antimicrobial and other health benefits of vinegar [22]. Regarding L-menthol, 4-ketoisophorone, and p-cymen-8-ol, they have not been previously reported in vinegar. Eucalyptol is usually present in wines from vineyards in the vicinity of eucalyptus forests [33]. Several studies have also suggested that it can be produced by the chemical transformation of limonene and α-terpineol and this may explain its presence in wines from vines not grown near eucalyptus trees [34]. However, its identification along with L-menthol and phoracanthal, which is a pheromone of Phoracantha spp. (eucalyptus borers), also correlates to eucalyptus trees. Since the Corinthian currants used in this study are cultivated away from the coast where eucalyptus trees grow, the presence of these compounds should be further investigated.

To conclude, comparing the different products, it can be observed that higher amounts of esters (~62% of identified compounds) were found in vinegar made with immobilized cells, than that made with free cells (41–55%). The percentages of esters found in the vinegar were 8 times higher than those identified in the raw material (FSS) [10]. On the other hand, higher percentages of alcohols (~28%) were identified in the vinegar made by free cells of the wild AAB culture (FCW), while all other vinegar samples and FSS had similar percentages (in the range 9–18%). The percentages of organic acids were similar in most samples (15–23%), except for the vinegar prepared by free cells of the selected, mixed culture (FCM) (43%). Higher percentages of carbonyl compounds (41%) were found in the volatile profile of FSS, while the vinegar contained carbonyl compounds at lower or similar percentages (1.5–2.0%), except for the FCM vinegar (6%). Terpenes were found at lower percentages in the vinegar products (0.21–0.38%) compared to FSS (with the exception of thujone), with a higher percentage being that of sample FCM. The lactones were also reduced (0.1–0.2%), with the highest percentage also being that of FCM. Finally, hydrocarbons were found at much lower percentages in the vinegar products (~0.1%) compared to FSS (7.6%), while for the miscellaneous compounds the highest percentage was again found in the FCM vinegar (~0.35).

## 4. Conclusions

The results of this study showed that it is possible to produce vinegar from sweet wine made by the alcoholic fermentation of aqueous extracts of the side stream generated from Corinthian currants (FSS), using a mixed selected culture (*A. aceti* and *K. europaeus*) and a wild AAB culture, free or immobilised on a natural cellulosic material. The cultures could be reused for repeated acetification batches, which is important for the functional stability of the process. However, the recovery of the biocatalyst is easier in the case of the immobilized cells, which remain in the bioreactor while the vinegar is easily decanted and has a better clarity compared to that made by free cells. The effect of cell immobilization on the acetification kinetics was more obvious in the case of the *A. aceti* and *K. europaeus* culture, as well as in the case of all cultures after storage for 2 months at 4 °C. Specifically, cold storage of all cultures reduced their activity during the first fermentation batch, but the immobilized cells, especially of the selected culture, performed better in the subsequent fermentation batches, indicating better survival of the immobilised bacteria during storage and readaptation to the acetification environment. The vinegar had a high antioxidant capacity and phenolic content and a rich volatilome. Higher percentages of esters on total volatiles were identified in vinegar made by immobilized cells, while higher percentages of alcohols were found in vinegar made by free cells of the wild culture. The results are encouraging for vinegar production from sweet FSS wine with immobilized cells of a mixed *A. aceti* (quick fermentation starter) and *K. europaeus* (acetic acid resistant) culture.

## Figures and Tables

**Table 1:** Kinetic parameters of the sweet FSS wine acetification process using the wild and the pure, mixed AAB cultures, free and immobilized on DCM.

Culture	Initial Sugar	Initial Ethanol	Acetification Time *	Final Acidity	Residual Sugar	Residual Ethanol
	(% *w*/*v*)	(% *v*/*v*)	(Days)	(g Acetic Acid/L)	(% *w*/*v*)	(% *v*/*v*)
Wild AAB culture						
FCW	2.61 ± 0.37 ^a^	4.73 ± 0.25 ^a^	>3	55.07 ± 2.39 ^a^	0.31 ± 0.06 ^a^	0.40 ± 0.53 ^a^
ICW	3.03 ± 0.17 ^b^	4.30 ± 1.22 ^a^	>5	49.54 ± 0.99 ^b^	1.48 ± 0.10 ^b^	0.90 ± 0.85 ^a^
FCW-2Μ	3.03 ± 0.03 ^a^	5.83 ± 1.84 ^a^	>20	39.97 ± 2.82 ^c^	0.50 ± 0.13 ^a^	0.78 ± 0.69 ^a^
ICW-2Μ	3.80 ± 0.22 ^b^	6.11 ± 1.41 ^a^	>10	47.44 ± 1.81 ^b^	1.47 ± 0.31 ^b^	1.56 ± 0.32 ^a^
Mixed AAB culture (*A. aceti* and *K. europaeus*)						
FCM	4.03 ± 0.46 ^b^	4.85 ± 0.19 ^a^	>9	49.00 ± 2.01 ^b^	1.65 ± 0.55 ^b^	1.39 ± 1.22 ^a^
ICM	3.88 ± 0.03 ^b^	4.12 ± 1.49 ^a^	>2	48.77 ± 1.83 ^b^	2.55 ± 0.21 ^c^	0.58 ± 0.43 ^a^
FCM-2Μ	4.03 ± 0.79 ^b^	4.75 ± 0.56 ^a^	>11	41.57 ± 0.81 ^c^	1.25 ± 0.09 ^b^	0.34 ± 0.42 ^a^
ICM-2Μ	4.05 ± 0.07 ^b^	5.93 ± 0.64 ^a^	>7	42.66 ± 7.32 ^b^	2.99 ± 0.06 ^d^	1.08 ± 0.19 ^a^

FCW, ICW: Free and immobilised cells of wild culture, respectively. FCM, ICM: Free and immobilised cells of selected, mixed culture, respectively. 2Μ: 2-Month storage at 4 °C. * Time when the acidity levels tend to reach a plateau. Different superscript letters in the same column indicate statistically significant differences.

**Table 2:** Total phenolic content (TPC) and antioxidant capacity (AC) of the vinegar made from FSS sweet wine using free and immobilized cells of the mixed *A. aceti* and *K. europaeus* culture.

Biocatalyst	TPC	AC
	(mg GAE/L)	(mg AAE/L)
Free cells	333.10 ± 11.98 ^a^	263.50 ± 8.41 ^a^
Immobilized cells	222.14 ± 2.86 ^b^	277.10 ± 6.66 ^a^

Different superscript letters in the same column indicate statistically significant differences.

**Table 3:** Volatiles identified by SPME GC/MS analysis (normalized peak areas %) in the Corinthian currants (*Vostitsa*) finishing side-stream (FSS), as well as in vinegar produced from FSS wine by free cells of wild vinegar culture (FCW) and mixed *A. aceti* and *K. europaeus* culture (FCM), and the corresponding immobilized cultures (ICW, ICM).

Compound	RID	RI_ref_	RI	FSS	FCW	FCM	ICW	ICM	Odour/Flavour Descriptions [35]	Occurrence [10,27,30,31,32,36,37,38,39,40,41,42,43,44,45,46]
Esters										
Ethyl formate	Β	824	819	<0.01	0.09	<0.01	0.05	0.04	Ethereal, green, rose, cognac, winey, fruity	W, O
Methyl acetate	A	828	820	0.20	0.04	0.12	0.09	0.06	Ethereal, estery, fruity, winey, cognac, rummy	W, O, VN
Ethyl acetate	A	888	882	5.30	46.20	30.12	32.38	36.86	Ethereal, fruity, grape, rummy	R, W, VN
Ethyl propanoate	A	953	949	<0.01	0.07	0.04	0.06	0.06	Fruity, winey, grape, rummy	W, VN
Ethyl 2-methylpropanoate (ethyl isobutyrate)	A	961	958	<0.01	0.09	0.06	0.10	0.11	Ethereal, fruity, alcoholic, fusel, rummy	W, VN
Propyl acetate	A	973	970	<0.01	0.04	<0.01	0.09	0.10	Solvent, pungent, fruity, banana, celery, honey	W, VN
2-Methylpropyl acetate (isobutyl acetate)	A	1012	1012	<0.01	0.25	0.19	0.97	0.66	Fruity, ethereal, tropical, banana	W, VN
Ethyl butanoate (ethyl butyrate)	A	1035	1034	<0.01	0.07	0.06	0.03	0.05	Fruity, pineapple, cognac	W, VN
Ethyl 2-methylbutanoate	B	1051	1051	<0.01	0.02	<0.01	0.02	0.02	Fruity, berry, apple, green	W, VN
Ethyl 3-methylbutanoate (ethyl isovalerate)	B	1068	1066	<0.01	0.29	0.44	0.59	0.39	Fruity, pineapple apple, orange	W, VN
Butyl acetate	A	1074	1070	<0.01	0.02	0.01	<0.01	0.02	Ethereal, solvent, fruity, banana	V, W, VN
3-Methylbutyl acetate (isoamyl acetate)	A	1122	1118	0.05	5.89	3.76	19.81	16.91	Fruity, banana, solvent	W, VN
Butyl butanoate (butyl butyrate)	B	1220	1217	<0.01	<0.01	0.03	0.01	0.01	Fruity, cherry, tropical, diffusive, ripe	O
Ethyl hexanoate (ethyl caproate)	A	1233	1229	0.07	0.08	0.06	0.36	0.71	Fruity, waxy, pineapple, banana	R, W, VN
Ethyl heptanoate (ethyl capronate)	B	1331	1336	<0.01	<0.01	<0.01	0.05	0.12	Fruity, pineapple, cognac, rummy, winey	W, VN
Ethyl 2-hydroxypropanoate (ethyl lactate)	A	1347	1344	0.02	<0.01	<0.01	0.01	<0.01	Sharp, fruity, pineapple, caramel, buttery	W, VN
Ethyl octanoate (ethyl caprylate)	A	1435	1435	0.70	0.06	0.06	0.46	0.94	Fruity, banana, pear, winey, brandy, waxy	R, W, VN
Ethyl 2-hydroxy-4-methylpentanoate	C	1547	1545	<0.01	0.04	0.01	0.01	0.01	Fresh, fruity, blackberry	W
Ethyl decanoate (ethyl caprate)	A	1638	1640	0.33	0.02	<0.01	0.01	0.04	Waxy, fruity, apple, grape, brandy	R, W, VN
Diethyl butanedioate (diethyl succinate)	A	1680	1681	<0.01	0.07	0.03	0.03	0.06	Fruity, cooked apple, ylang	R, W, VN
Phenylmethyl acetate (benzyl acetate)	Β	1720	1726	<0.01	<0.01	<0.01	0.02	0.01	Floral, fruity, jasmine, balsamic	R, O, VN
Ethyl 2-phenylacetate (ethyl benzeneacetate)	C	1783	1781	<0.01	0.33	0.16	0.14	0.17	Floral, rose, honey, cocoa, balsamic, anisic	R, W, VN
2-Phenylethyl acetate	A	1813	1810	0.38	1.59	5.94	6.23	4.68	Floral, rose, honey, fruity	R, W, VN
2-Phenylethyl butanoate (phenethyl butyrate)	B	1958	1965	0.02	<0.01	<0.01	<0.01	<0.01	Floral, strawberry, musty, yeasty	W
Ethyl 9-decenoate	B	1694	1695	<0.01	<0.01	<0.01	0.03	0.04	Fruity, fatty	W
Octyl octanoate	B	2009	2014	<0.01	0.01	0.05	0.05	0.03	Coconut, oily, fruity	O
Total	26			<7.24	<55.33	<41.23	<61.62	<62.12		
Alcohols										
Ethanol	A	932	931	6.55	13.29	1.63	5.06	8.89	Alcoholic, strong, ethereal, medicinal	W, VN
2-Methyl-1-propanol (isobutanol)	A	1092	1098	0.23	0.12	0.01	0.11	0.11	Ethereal, winey, fusel, whiskey	R, W, VN
1-Butanol	A	1142	1151	0.12	0.02	<0.01	<0.01	<0.01	Fusel, oily, balsamic, whiskey, banana	R, W
1-Penten-3-ol (ethyl vinyl carbinol)	B	1159	1166	0.25	<0.01	<0.01	<0.01	<0.01	Ethereal, fruity, tropical, horseradish	O, W
3-Methyl-1-butanol (isoamyl alcohol)	A	1209	1211	1.58	7.60	0.97	3.20	3.62	Fusel, oil, whiskey, fruity, banana, cognac	R, W, VN
1-Pentanol	A	1250	1256	0.59	<0.01	<0.01	<0.01	<0.01	Pungent, fermented, fusel, winey, balsamic	R, W, VN
(Z)-2-Penten-1-ol	B	1318	1324	0.08	<0.01	<0.01	<0.01	<0.01	Phenolic, fruity, medicinal, metallic	W
3-Methyl-2-buten-1-ol (prenol)	C	1320	1324	0.05	<0.01	<0.01	<0.01	<0.01	Fruity, green, lavender	R
1-Hexanol	A	1355	1357	1.31	0.01	<0.01	<0.01	0.01	Pungent, ethereal, fusel, fruity, alcoholic	V, R, W, VN
(Z)-3-Hexen-1-ol	B	1382	1387	0.09	<0.01	<0.01	<0.01	<0.01	Fresh, grassy, herbal, oily, pungent	R, VN
(E)-2-Hexen-1-ol	B	1405	1409	0.09	<0.01	<0.01	<0.01	<0.01	Fresh, herbal, leafy, fruity, banana, fatty	R, V
1-Octen-3-ol	A	1450	1454	2.00	<0.01	<0.01	0.01	0.01	Mushroom, earthy, oily, umami, chicken	V, R, W
1-Heptanol	B	1453	1460	0.14	<0.01	<0.01	0.01	0.02	Solvent, leafy, woody, musty, nutty, oily	R, W
2-Ethyl-1-hexanol	A	1491	1493	0.62	1.36	0.58	0.19	0.20	Citrus, fresh, floral, fruity, oily	R, V, W, VN
(E)-2-Hepten-1-ol	C	1517	1514	0.06	<0.01	<0.01	<0.01	<0.01	Green, pungent, fatty, plastic	V
2,3-Butanediol isomer 1	C	1543	1544	0.49	0.06	0.11	0.05	0.06	Odourless, fruity, creamy, buttery	R, W, VN
1-Octanol	A	1557	1562	1.07	0.06	0.05	0.01	0.02	Waxy, citrus, aldehydic, floral	R, V, W, VN
2,3-Butanediol isomer 2	C	1556	1581	0.53	0.02	0.02	<0.01	0.02	Odourless, fruity, creamy, buttery	R, W
(E)-2-Octen-1-ol	C	1614	1617	0.16	<0.01	<0.01	<0.01	<0.01	Green, citrus, vegetable, fatty, oily, fruity	R
2-Furanmethanol (furfuryl alcohol)	B	1660	1661	0.11	<0.01	<0.01	<0.01	<0.01	Sulphuraceous, estery, alcoholic, musty, caramel, bready, coffee, burnt	O, W, VN
1-Nonanol	B	1660	1665	0.68	<0.01	0.01	<0.01	0.02	Fresh, rose, orange, fatty, aldehydic, spicy	R, V, W, VN
2-Dodecanol	C	1813	1822	0.14	0.01	<0.01	<0.01	<0.01	n/f	W
Phenylmethanol (benzyl alcohol)	B	1870	1875	0.54	<0.01	<0.01	0.01	0.02	Floral, rose, fruity, cherry, almond, balsamic, bitter	R
2-Phenylethanol (phenylethyl alcohol)	A	1906	1912	0.63	5.47	5.66	4.71	4.92	Floral, rose, fresh, bready, honey	V, R, W, VN
1-Tetradecanol (myristyl alcohol)	C	2165	2181	0.42	0.04	0.05	0.01	0.03	Fruity, orris, coconut, waxy	R
Total	25			<18.53	<28.19	<9.24	<13.51	<18.06		
Organic acids										
Acetic acid	A	1449	1445	10.78	14.04	38.67	19.80	16.60	Sharp, pungent, vinegar, overripe fruit	W, VN
Formic acid	B	1503	1506	0.05	<0.01	<0.01	<0.01	<0.01	Sharp, acetic, fruity, astringent, bready, mustard	O
Propanoic acid	B	1535	1538	0.08	<0.01	0.04	0.02	0.01	Pungent, acidic, fatty, cheesy, vinegar, fruity	W, V, VN
2-Methylpropanoic acid (isobutyric acid)	C	1570	1568	<0.01	0.05	0.20	0.09	0.05	Acidic, cheesy, dairy, buttery, rancid	R, W, VN
Butanoic acid	B	1625	1628	0.01	0.02	0.10	<0.01	<0.01	Sharp, acetic, dairy, cheesy, buttery, fruity	R, W, VN
3-Methylbutanoic acid (isovaleric acid)	B	1666	1670	0.14	0.95	3.41	1.94	0.60	Acidic, pungent, dairy, waxy, fruity	R, W, VN
2-Methylbutanoic acid	C	1662	1671	0.05	0.10	0.28	0.08	<0.01	Fruity, pungent, dairy, buttery, cheesy	W
Pentanoic acid (valeric acid)	B	1733	1737	0.12	<0.01	<0.01	0.01	0.01	Acidic, cheesy, sour milk, tobacco, fruity	R, V, W
Hexanoic acid (caproic acid)	A	1846	1844	1.14	0.06	0.21	0.21	0.25	Sour, sweat, fatty, cheesy, fruity, phenolic	R, W, VN
3-Methylhexanoic acid	C	-	1954	0.16	<0.01	0.02	0.02	0.04	n/f	n/f
Octanoic acid (caprylic acid)	A	2060	2063	8.50	<0.01	<0.01	0.59	0.60	Fatty, waxy, rancid, oily, cheesy, brandy	R, W, VN
Nonanoic acid	C	2171	2174	0.23	0.02	0.02	0.02	0.02	Fatty, dirty, waxy, cheesy, creamy	V, R, VN
n-Decanoic acid (capric acid)	Β	2276	2251	0.89	<0.01	0.02	0.06	0.10	Rancid, sour, fatty, citrus, soapy, fruity	R, W, VN
Total	13			<22.16	<15.30	<43.00	<22.86	<18.31		
Carbonyl compounds										
Acetaldehyde	A	702	701	<0.01	0.11	0.08	0.26	0.24	Pungent, ethereal, aldehydic, fruity	O, W
2-Methylpropanal (isobutyraldehyde)	B	819	13	0.14	<0.01	<0.01	<0.01	<0.01	Pungent, aldehydic, floral, herbal, malty	O, V
Butanal (butyraldehyde)	B	877	867	0.03	<0.01	<0.01	<0.01	<0.01	Pungent, cocoa, musty, malty, fermented	O
2-Butanone (methyl ethyl ketone)	B	907	899	0.04	<0.01	<0.01	<0.01	<0.01	Acetone, ethereal, fruity, camphoraceous	O
2-Methylbutanal	B	914	908	0.59	<0.01	0.02	0.01	<0.01	Musty, rummy, malty, fermented, cocoa, coffee, nutty, caramel, fruity	O, W
3-Methylbutanal (isovaleraldehyde)	B	918	911	1.64	0.02	0.05	0.02	0.01	Ethereal, aldehydic, cocoa, peach, fatty, fruity, nutty, leafy	O, V, W
3-Methyl-2-butanone	Β	936	925	<0.01	<0.01	<0.01	0.04	0.04	n/f	n/f
2,3-Butanedione (diacetyl)	A	979	970	5.93	0.03	0.46	0.06	0.03	Buttery, creamy, pungent, caramel, milky	R, W, VN
Hexanal	A	1083	1076	3.33	0.06	0.15	0.01	0.01	Fresh, fatty, aldehydic, grassy, fruity, woody	V, R, W
2-Methyl-2-butenal	B	1095	1089	<0.01	<0.01	<0.01	0.04	0.03	Diffusive, green, ethereal, pungent, nutty, anisic	O, VN
2-Heptanone	B	1182	1178	0.05	<0.01	<0.01	0.01	<0.01	Fruity, spicy, ketonic, waxy, herbal, coconut, woody, banana	O, V, W, VN
Heptanal (oenanthic aldehyde)	B	1184	1179	0.17	<0.01	<0.01	<0.01	<0.01	Fresh, aldehydic, fatty, herbal, cognac, ozone, clover, cilantro	O, V, R, VN
3-Hydroxy-2-butanone (acetoin)	A	1284	1281	21.58	0.24	1.85	0.22	0.17	Fatty, oily, buttery, creamy, milky, yoghurt	R, W, VN
Octanal	B	1289	1284	0.21	<0.01	0.01	0.01	<0.01	Aldehydic, waxy, fatty, citrus, orange peel	V, R, W, VN
1-Hydroxy-2-propanone (hydroxyacetone)	B	1303	1293	<0.01	<0.01	0.01	<0.01	<0.01	Pungent, sweet, caramel, ethereal, burnt	O
2-Heptenal	B	1323	1319	0.21	<0.01	<0.01	<0.01	<0.01	Green, fatty	V, R, VN
6-Methyl-5-hepten-2-one	C	1338	1335	0.09	<0.01	<0.01	<0.01	<0.01	Fruity, apple, musty, ketonic, creamy	R, W
3-Acetoxy-2-butanone (acetoin acetate)	C	1378	1381	0.10	0.01	0.17	0.02	0.01	Sweet, fruity, chemical, pineapple, banana, rummy, grape, winey	n/f
1-Hydroxy-2-butanone	B	1388	1376	0.02	<0.01	<0.01	<0.01	<0.01	Sweet, coffee, musty, oily	n/f
2-Nonanone	C	1390	1387	<0.01	<0.01	<0.01	0.12	0.05	Fresh, weedy, earthy, cheesy, fruity	V, W, VN
Nonanal	B	1391	1391	0.41	0.01	0.02	0.02	0.02	Waxy, aldehydic, rose, orange peel, effervescent	V, R, W
5-Ethylcyclopentene-1-carbaldehyde (Phoracanthal)	C	1410	1411	0.40	<0.01	<0.01	0.01	0.01	n/f	Pheromone
3-Octen-2-one	C	1411	1405	0.31	<0.01	<0.01	<0.01	<0.01	Earthy, spicy, creamy, oily, herbal, mushroom, hay, blueberry	V, R
(E)-2-Octenal	C	1429	1426	0.16	<0.01	<0.01	<0.01	<0.01	Fresh, spicy, oily, fatty, waxy, cucumber, herbal, banana, brothy	O, R, VN
2-Furfuraldehyde (furfural)	A	1461	1459	3.37	0.35	0.74	0.19	0.16	Woody, bready, nutty, caramel, burnt, astringent	O, V, R, W, VN
Phenylmethanal (benzaldehyde)	A	1520	1517	0.96	0.31	2.17	0.71	0.47	Sharp, oily, bitter, almond, cherry, nutty, woody	V, R, W, VN
(E)-2-Nonenal	C	1534	1533	0.07	<0.01	<0.01	<0.01	<0.01	Fatty, soapy, aldehydic, cucumber, citrus, melon	V, R, W
(3E,5E)-3,5-Octadien-2-one	C	1570	1569	0.12	<0.01	<0.01	<0.01	<0.01	Fruity, grassy	V
5-Methyl-2-furfural	B	1570	1572	0.25	0.03	0.09	0.01	0.01	Spicy, caramel, maple, grain	R, W, VN
6-Methyl-3,5-heptadiene-2-one	B	1602	1591	0.27	<0.01	<0.01	<0.01	<0.01	Cinnamon, coconut, spicy, woody, weedy	V, R
Ethyl-1H-pyrrole-2-carboxaldehyde	C	1610	1605	0.14	<0.01	<0.01	<0.01	<0.01	Burnt, roasted, smoky	R
1-Phenylethanone (acetophenone)	B	1647	1646	<0.01	<0.01	0.02	0.03	0.03	Sweet, pungent, bitter, coumarinic, almond	R, V, VN
2,4-Nonadienal	C	1700	1700	0.07	<0.01	<0.01	<0.01	<0.01	Fatty, cucumber, melon, chicken, citrus peel	O, V, R
2-Acetylpyrrole	B	1973	1971	0.03	<0.01	<0.01	<0.01	<0.01	Sweet, musty, nutty	W
2-Hydroxyacetylfuran	C	1995	2003	0.15	<0.01	<0.01	<0.01	<0.01	n/f	W
2,4-Decadienal	B	1797	1805	0.10	<0.01	<0.01	<0.01	<0.01	Fresh, citrus, orange, fatty, chicken, fried	O, V
1H-Pyrrole-2-carboxaldehyde (pyrrole aldehyde)	B	2030	2023	0.01	<0.01	<0.01	<0.01	<0.01	Ethereal, musty, beefy, coffee	O
Total	37			<41.01	<1.44	<6.07	<1.98	<1.51		
Terpenes										
D-Limonene (1-methyl-4-prop-1-en-2-ylcyclohexene)	A	1200	1185	0.39	0.06	0.20	0.11	0.09	Citrus, herbal, terpenic, camphoraceous	V, R, W, VN
Eucalyptol (1,8-Cineole; 1,3,3-trimethyl-2-oxabicyclo [2.2.2]octane)	B	1213	1200	<0.01	<0.01	0.01	<0.01	<0.01	Minty, herbal, eucalyptus, camphoraceous, cooling	V, W, VN
Thujone [(1S,4S,5R)-4-methyl-1-propan-2-ylbicyclo [3.1.0]hexan-3-one]	B	1430	1416	<0.01	0.04	0.06	0.03	0.03	Cedar leaf, thujonic, menthol	O
Linalool (3,7-dimethyl-1,6-octadien-3-ol)	A	1547	1551	0.08	<0.01	<0.01	0.02	0.02	Citrus, floral, woody, green	V, R, W
β-Cyclocitral (2,6,6-trimethylcyclohexene-1-carbaldehyde)	C	1611	1618	0.08	<0.01	<0.01	<0.01	<0.01	Medicinal, phenolic, tropical, saffron, herbal, rose, tobacco, fruity, leathery	O, V
L-Menthol (5-methyl-2-(1-methylethyl)cyclohexanol)	C	1633	1647	<0.01	<0.01	<0.01	0.02	0.02	Minty, cooling, spicy, camphoraceous	O, W
4-Ketoisophorone (2,6,6-trimethyl-2-cyclohexene-1,4-dione)	C	1676	1690	0.04	<0.01	<0.01	<0.01	<0.01	Musty, woody, sweet, tea, citrus, floral	W
α-Terpineol [2-(4-methyl-3-cyclohexen-1-yl)-2-propanol]	A	1697	1700	0.07	<0.01	<0.01	0.03	0.02	Pine, woody, resinous, lemon, floral, soapy	V, R, W, VN
L-Borneol (1,7,7-trimethyl-bicyclo[2.2.1]heptan-2-ol)	B	1702	1706	<0.01	<0.01	<0.01	0.01	<0.01	Pine, woody, musty, camphor, peppery, earthy	O, V
β-Damascenone (1-(2,6,6-trimethylcyclohexa-1,3-dien-1-yl)but-2-en-1-one)	C	1823	1818	0.06	<0.01	0.01	0.02	0.02	Fruity, rose, plum, grape, woody, tobacco	V, R, W, VN
p-Cymen-8-ol (2-(4-methylphenyl)propan-2-ol)	C	1852	1851	<0.01	<0.01	0.01	0.01	0.01	Sweet, fruity, floral, coumarinic, camphoraceous	O
*trans*-Geranylacetone [(E)-6,10-dimethylundeca-5,9-dien-2-one]	C	1859	1853.9	0.09	<0.01	0.02	0.02	0.02	Fresh, fruity, floral, tropical, waxy, woody, rose, magnolia, pear, apple, banana	V, R, W
*trans*-β-Ionone [(E)-4-(2,6,6-trimethyl-1-cyclohexen-1-yl)-3-buten-2-one]	C	1940	1941	0.08	<0.01	<0.01	<0.01	<0.01	Powdery, floral, fruity, woody, orris, berry, seedy	V, R, W, VN
Total	13			<0.94	<0.21	<0.38	<0.31	<0.28		
Lactones										
γ-Butyrolactone (dihydrofuran-2(3H)-one)	B	1632	1621	1.92	0.02	0.05	0.01	0.02	Creamy, oily, caramel, milky, fruity, peach	V, R, W, VN
β-Angelica lactone (5-methyl-2(5H)-furanone)	C	1669	1674	0.03	<0.01	<0.01	<0.01	<0.01	Coconut, nutty, coumarinic, vanilla, hay, solvent, oily, tobacco, creamy	R, W
γ-Hexalactone (dihydro-5-ethyl-2(3H)-furanone)	C	1694	1698	0.04	<0.01	<0.01	<0.01	<0.01	Coconut, woody, sweet, creamy	V, R
2-hexen-1,4-lactone (5-ethyl-2(5H)-furanone)	C	1745	1753	0.06	<0.01	<0.01	<0.01	<0.01	Spice	n/f
γ-Octalactone (5-butyldihydrofuran-2(3H)-one)	C	1910	1915	0.01	<0.01	<0.01	<0.01	<0.01	Sweet, creamy, coconut, milky, soapy, fruity, peach, roasted	O, W, VN
δ-Octalactone (6-propyltetrahydro-2H-pyran-2-one)	C	1976	1978	<0.01	<0.01	0.03	0.03	0.04	Fatty, coconut, tonka, tropical, dairy, fruity	O, W
γ-Nonalactone (dihydro-5-pentyl-2(3H)-furanone)	C	2024	2028	0.06	0.01	0.08	0.03	0.03	Coconut, creamy, waxy, buttery, milky	R, W, VN
Total	7			<2.13	<0.08	<0.20	<0.11	<0.13		
Miscellaneous compounds										
Dimethyl sulphide	B	754	739	0.03	<0.01	0.01	0.02	0.01	Sulphuraceous, vegetable, onion, cabbage, asparagus, creamy, molasses, minty	W
2-Ethylfuran	B	950	943	0.04	<0.01	<0.01	<0.01	<0.01	Burnt, earthy, malty, solvent-like, musty	O
1,4-Dioxane (IS)			1055.5							
2-Pentylfuran	B	1231	1220	0.22	<0.01	<0.01	<0.01	<0.01	Fruity, earthy, beany, vegetable, metallic, waxy, musty, caramel	R
2,6-Dimethylpyrazine	B	1328	1327	<0.01	0.02	0.05	<0.01	<0.01	Cocoa, roasted, coffee, nutty, musty, bready	R, O, VN
2-Ethyl-6-methylpyrazine	B	1386	1385	0.04	<0.01	<0.01	<0.01	<0.01	Roasted potato, roasted hazelnut	R
2,3,5-Trimethylpyrazine	B	1402	1404	<0.01	0.03	0.11	0.01	0.01	Nutty, musty, potato	O, VN
2,3-Dimethyl-5-ethylpyrazine	C	1460	1463	0.08	<0.01	<0.01	<0.01	<0.01	Burned popcorn, roasted cocoa	O
2,3,5,6-Tetramethylpyrazine (Ligustrazine)	B	1469	1477	0.03	<0.01	<0.01	<0.01	<0.01	Nutty, musty, vanilla, earthy, cocoa, peanut, coffee, asparagus	O, VN
2-Acetylfuran (2-furyl methyl ketone)	B	1499	1500	0.16	0.01	0.04	0.01	0.01	Balsamic, almond, cocoa, coffee, caramel, roasted, nutty, milky, lactonic	R, VN
Benzothiazole	C	1958	1953	<0.01	<0.01	0.09	0.01	<0.01	Sulphuraceous, vegetable, cooked, nutty, meaty	V, W, VN
Total	11			<0.63	<0.13	<0.35	0.11	<0.10		
Hydrocarbons (alkanes/alkenes)										
Hexane	A	600	600	5.91	0.01	0.02	<0.01	<0.01	Gasoline-like, petrolic	O
Heptane	A	700	700	0.35	<0.01	<0.01	<0.01	<0.01	Sweet, ethereal, petrolic	O
Octane	A	800	800	0.33	<0.01	<0.01	<0.01	<0.01	Gasoline-like	O, V
Nonane	A	900	900	0.07	<0.01	<0.01	<0.01	<0.01	Gasoline-like	O, V
Decane	A	1000	999	0.10	<0.01	<0.01	<0.01	<0.01	Gasoline-like	V, W
Dodecane	A	1200	1200	0.32	<0.01	<0.01	<0.01	<0.01	Gasoline-like to odourless	O
Tetradecane	A	1400	1400	0.46	<0.01	<0.01	<0.01	<0.01	Gasoline-like to odourless	R, VN
Hexadecane	A	1600	1600	0.06	<0.01	<0.01	<0.01	<0.01	Gasoline-like to odourless	O, R, VN
Total	8			7.60	<0.08	<0.09	<0.08	<0.08		

RID: Reliability of Identification; RI: Retention Index; R: Raisins; W: Wine; V: Parts of the vine plant or grapes; VN: Vinegar; O: Other plant source; n/f: Not found.

## Supplementary Materials

The following are available online at https://www.mdpi.com/article/10.3390/foods10123133/s1, Figure S1: Kinetics of the acetification of wine at 30 °C using wild AAB culture, free (FCW) and immobilized (ICW), and the mixed AAB culture (*A. aceti* & *K. europaeus*), free (FCM) and immobilized (ICM), Figure S2: Kinetics of the acetification of wine at 30 °C, reusing the wild AAB culture, free (FCW-2Μ) and immobilized (ICW-2Μ), and the mixed AAB culture (*A. aceti* & *K. europaeus*), free (FCM-2Μ) and immobilized (ICM-2Μ), after 2-months of cold storage at 4 °C. Table S1: Parameters of the sigmoidal (Boltzmann) model curve fit of the experimental data means of 3 acetification batches (Figure 3 and Figure 4), Table S2. Acetification rates as slopes of the linear fits of the growth phases of the Boltzmann sigmoidal curves of Figure 3 and Figure 4.
