# Supplementary material for: Vinegar Production from Corinthian Currants Finishing Side-Stream: Development and Comparison of Methods Based on Immobilized Acetic Acid Bacteria
Source: Foods. 2021 Dec 17;10(12):3133. doi: 10.3390/foods10123133 (PMC8700812; doi:10.3390/foods10123133)
Supplement: Supplementary file 1 [file foods-10-03133-s001.zip › foods-1479196-supplementary.pdf]

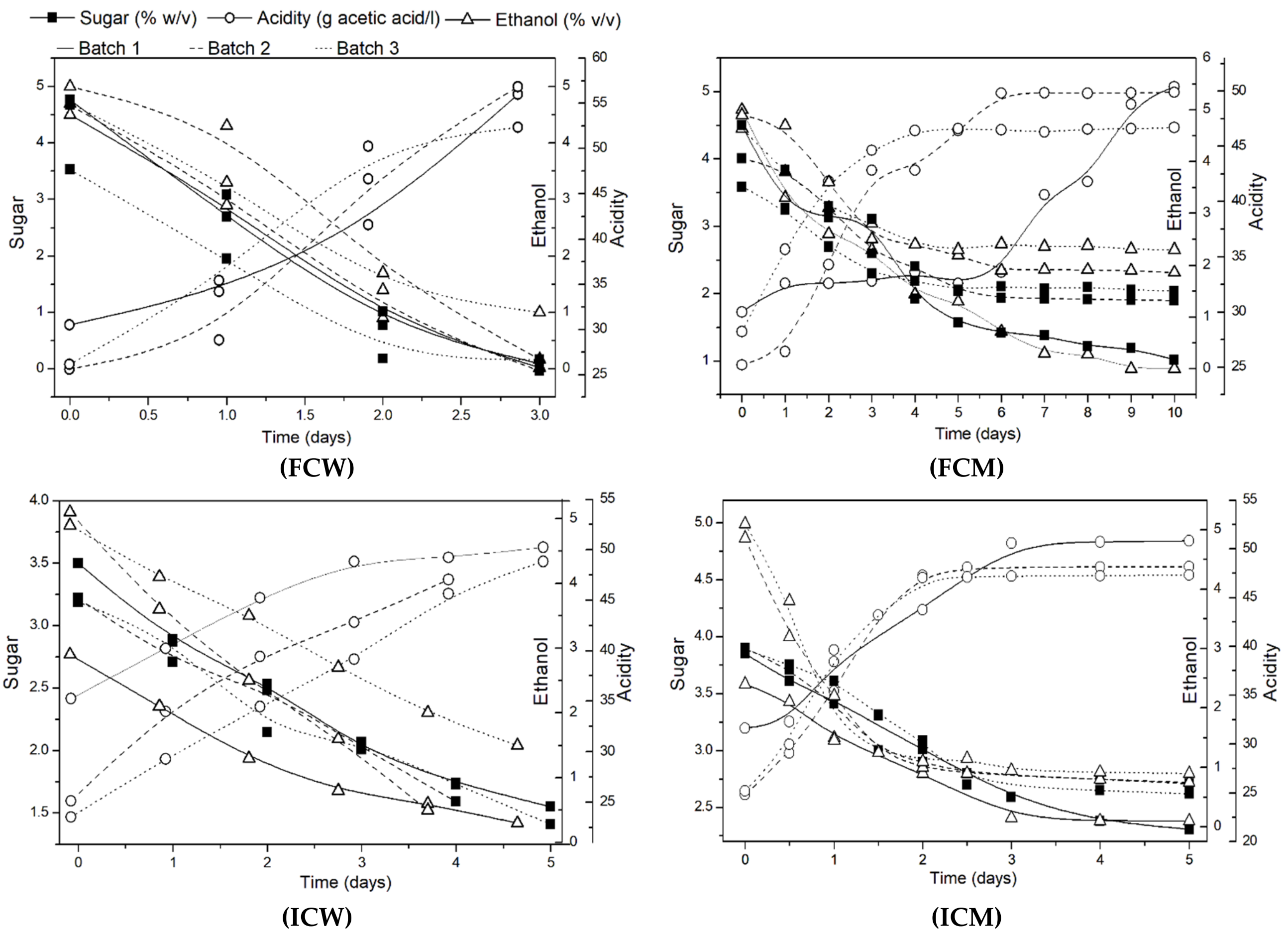

Figure S1. Kinetics of the acetification of wine at 30ºC using wild AAB culture, free (FCW) and immobilized (ICW), and the mixed AAB culture (*A. aceti* & *K. europaeus*), free (FCM) and immobilized (ICM).

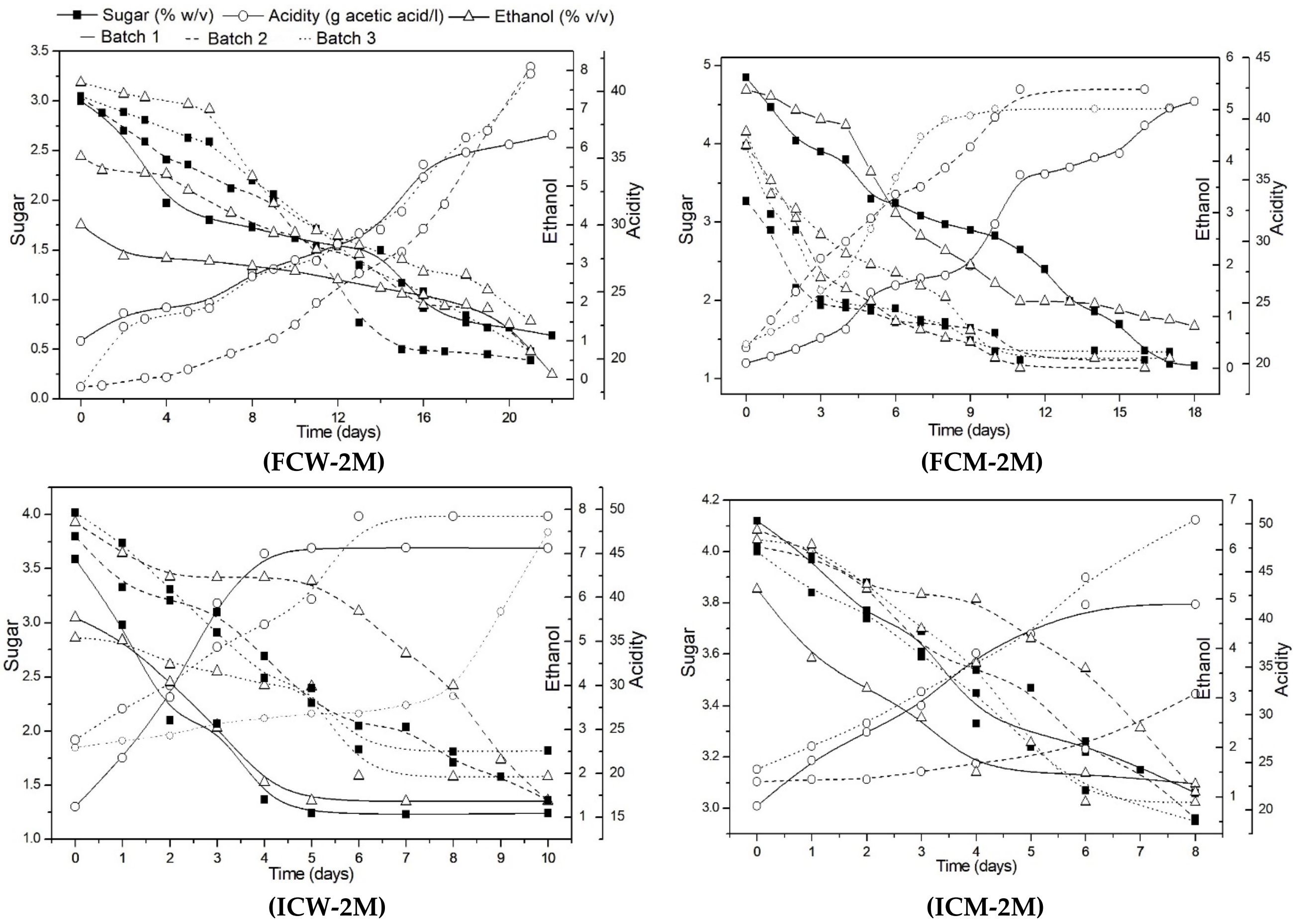

Figure S2. Kinetics of the acetification of wine at 30°C, reusing the wild AAB culture, free (**FCW-2M**) and immobilized (**ICW-2M**), and the mixed AAB culture (*A. aceti* & *K. europaeus*), free (**FCM-2M**) and immobilized (**ICM-2M**), after 2-months of cold storage at 4°C.

Table S1. Parameters of the sigmoidal (Boltzmann) model curve fit of the experimental data means of 3 acetification batches (Figures 3 and 4).

**Parameter**	**Biocatalyst**							
	**FCW**	**FCM**	**ICW**	**ICM**	**FCW-2M**	**FCM-2M**	**ICW-2M**	**ICM-2M**
Sugar								
A_1	2.9133	4.649	6.267	4.097	4.874	122.791	5.143	4.204
A_2	0.246	1.677	0.684	2.588	-0.541	0.911	1.432	2.866
t_o	1.071	1.732	-0.397	1.123	6.543	-29.864	1.206	3.425
dt	0.522	1.442	2.969	0.627	9.938	8.056	2.137	1.925
R^2	**1.000**	**0.9939**	**0.9988**	**0.9901**	**0.9990**	**0.9843**	**0.998**	**0.9977**
Ethanol								
A_1	5.0262	10.706	6.203	33.218	9.014	6.699	10.921	8.499
A_2	0.200	1.376	0.516	0.711	-15.217	0.290	-0.800	-0.398
t_o	1.394	-1.091	0.965	-1.648	39.385	2.507	2.101	2.865
dt	0.511	2.092	1.447	0.786	23.268	3.161	5.718	3.187
R^2	**1.000**	**0.9997**	**0.9964**	**0.9127**	**0.9554**	**0.9964**	**0.9985**	**0.9997**
Acidity								
A_1	26.163	-156.22	-9.246	22.421	18.746	16.379	-65.615	16.352
A_2	57.618	52.453	54.448	48.618	56.593	41.948	58.559	51.667
t_o	1.703	-9.754	-0.804	0.865	19.416	4.938	-5.564	4.453
dt	0.534	4.845	2.336	0.492	6.102	3.254	6.752	2.960
R^2	**1.000**	**0.9962**	**0.9996**	**0.9978**	**0.9909**	**0.9995**	**0.9968**	**0.9944**

Curve fit was done by Microcal Origin 6.0 software, based on the Boltzmann equation:

$$C(t) = \frac{A1 - A2}{1 + e^{\frac{t-to}{dt}}} + A2$$

where C(t) is the concentration of sugar, ethanol, or acidity in time t, *A1* and *A2* are the initial and final positions of two asymptotes to the *C(t)* curve, t_o is the t-coordinate of the highest value of the curve slope, and *dt* is the width of the step of exponential decrease.

Table S2. Acetification rates as slopes of the linear fits of the growth phases of the Boltzmann sigmoidal curves of Figures 3 and 4.

Linear fit*	Biocatalyst							
	FCW	FCM	ICW	ICM	FCW-2M	FCM-2M	ICW-2M	ICM-2M
Slope (b)	9.48±0.80	3.04±0.32	4.48±0.27	10.05±0.99	1.01±0.05	1.67±0.04	2.53±0.08	2.62±0.13
R^2	1.00±0.22	0.97±0.33	1.00±0.22	0.99±0.64	0.98±0.82	1.00±0.17	1.00±0.28	0.99±0.18

A=a+b×t; A=acidity (g/l), t=time (d)
